# Regenerative Medicine for Epilepsy: From Basic Research to Clinical Application

**DOI:** 10.3390/ijms141223390

**Published:** 2013-11-28

**Authors:** Takao Yasuhara, Takashi Agari, Masahiro Kameda, Akihiko Kondo, Satoshi Kuramoto, Meng Jing, Tatsuya Sasaki, Atsuhiko Toyoshima, Susumu Sasada, Kenichiro Sato, Aiko Shinko, Takaaki Wakamori, Yu Okuma, Yasuyuki Miyoshi, Naoki Tajiri, Cesario V. Borlongan, Isao Date

**Affiliations:** 1 Department of Neurological Surgery, Okayama University Graduate School of Medicine, 2-5-1, Shikata-cho, Okayama 700-8558, Japan; E-Mails: agarit@cc.okayama-u.ac.jp (T.A.); mrkameda@gmail.com (M.K.); domacv@yahoo.co.jp (A.K.); kuramoto1218@yahoo.co.jp (S.K.); dr.j_meng@hotmail.com (M.J.); tatu_tatu_sasa@yahoo.co.jp (T.S.); atsuhiko.t@kyj.biglobe.ne.jp (A.T.); sasadaminami@yahoo.co.jp (S.S.); satoken645@gmail.com (K.S.); shinkoaiko@gmail.com (A.S.); wakagon@cc.okayama-u.ac.jp (T.W.); y8u2bear4@hotmail.com (Y.O.); ya-miyo1@gold.megaegg.ne.jp (Y.M.); idate333@md.okayama-u.ac.jp (I.D.); 2 Department of Neurosurgery, University of South Florida College Medicine, 12901 Bruce B Downs Blvd, Tampa, FL 33612, USA; E-Mails: ntajiri@health.usf.edu (N.T.); cborlong@health.usf.edu (C.V.B.)

**Keywords:** brain-derived neurotrophic factor (BDNF), electrical stimulation, erythropoietin, GABAergic neuron, kainic acid, kindling, neural stem cell

## Abstract

Epilepsy is a chronic neurological disorder, which presents with various forms of seizures. Traditional treatments, including medication using antiepileptic drugs, remain the treatment of choice for epilepsy. Recent development in surgical techniques and approaches has improved treatment outcomes. However, several epileptic patients still suffer from intractable seizures despite the advent of the multimodality of therapies. In this article, we initially provide an overview of clinical presentation of epilepsy then describe clinically relevant animal models of epilepsy. Subsequently, we discuss the concepts of regenerative medicine including cell therapy, neuroprotective agents, and electrical stimulation, which are reviewed within the context of our data.

## 1. Introduction

Epilepsy is a chronic neurological disorder, characterized by various forms of seizures [1]. Antiepileptic drugs are the first treatment of choice for epilepsy patients. However, approximately 20%–40% of patients with epilepsy do not respond to treatment with antiepileptic drugs and are consequently considered as presenting with “refractory epilepsy” [2]. In some patients with temporal lobe epilepsy (TLE) accompanied by mesial temporal sclerosis or epilepsy likely induced by localized lesion, such as brain tumors, surgical resection is preferred [3]. For patients with refractory epilepsy, who do not respond well to surgery, various therapies, including electrical/magnetic stimulation, ketonic diet therapy, medication using adrenocorticotropic hormone or immunoglobulin, and behavioral therapy are effective to some extent [4]. However, many patients still suffer from intractable seizures, despite multimodal combination of currently existing therapies. Novel and efficacious therapeutic options are needed for patients with intractable seizures [5]. Stem cell therapy stands as an alternative treatment for epilepsy [6]. The use of induced pluripotent stem cells (iPS cells), especially when harvested from the epileptic patients themselves, may reveal new pathological mechanisms, as well as therapeutic targets for epilepsy [7]. Moreover, iPS cells pose as good donor cell candidates for transplantation, but only after overcoming logistical obstacles related to potential iPS tumorigenesis [8,9] and “seizure” activity possibly inherent in epileptic patient-derived stem cells.

In this article, the potential of regenerative medicine for epilepsy is reviewed. We initially review rodent models of epilepsy then subsequently highlight the research progress in cell therapy, neuroprotection, and electrical stimulation and whenever possible discuss our data relevant to these topics.

## 2. Basic Research and Clinical Application for Epilepsy

### 2.1. Rodent Models of Epilepsy, Epileptogenesis, and Neurogenesis

Rodent models of TLE have been widely explored. The pathogenesis of epilepsy and animal models are elegantly reviewed in the article by Sharma [10]. There are 2 major hypotheses on epileptogenesis, namely, recurrent excitation and recurrent inhibition hypothesis. The former holds the view that seizures result from hyperexcitability of dentate granule cells due to the abnormal excitatory circuitry mainly induced by mossy fiber sprouting. On the other hand, the latter adheres to the notion that the loss of inhibitory neurons makes epileptogenic foci in dentate granule cells. These two competing hypotheses are equally supported by many studies generated from both human patients and animal models.

In order to reveal the mechanisms of TLE, various methods have been explored using chemoconvulsants, electrical stimulations, tetanus toxins, hyperthermia, trauma, hypoxia for newborn animals, and genetic manipulations. Two different models of TLE that have been commonly used in the scientific community include the post-status epilepticus models and kindling models. In post-status epilepticus models, a severe insult (status epilepticus for human) results in spontaneous seizures after some latent period, usually for a few weeks. Kainic acid (KA) and pilocarpine [11,12] are two common chemoconvulsants used to induce post-status epilepticus model in rodents. Intraperitoneal and intrahippocampal injections of KA or pilocarpine have been employed to create the TLE model. Systemic administration is practical but leads to high mortality, whereas the stereotaxic injection of the chemoconvulsants requires surgical maneuvers; the animal mortality is low. The chemoconvulsant models exhibit several features of TLE, but electrical stimulation of the perforant pathway may be a superior model because of its lower mortality, higher success rate, and closer resemblance between this animal model and epilepsy patients [13]. For kindling models, the animal is usually exposed repeatedly to a mild electrical stimulation. The brain targets for the electrical stimulation include hippocampus, amygdala, or other hot regions implicated in the clinic as origins of epileptic seizures. Recognizing each animal model’s advantages and limitations will allow a better platform of investigations for epilepsy. These animal models have been used to study the pathological mechanisms of epilepsy and the evaluation of therapeutic potentials of regenerative medicine, which are discussed below.

### 2.2. Regenerative Medicine

#### 2.2.1. Cell Therapy

Recent progress in cell transplantation using the TLE model is well described by Shetty [4]. The TLE model resembles many of the patients’ pathological conditions, including cell loss of excitatory neurons in discreet hippocampal regions, decrease of inhibitory gamma-aminobutyric acid positive (GABAergic) interneurons, formation of abnormal neuronal circuit and changes in expression level of several receptors and ion channels. Finally the balance between excitation and inhibition in the hippocampus is disrupted with subsequent hyperexcitability of the hippocampus. In order to replace damaged hippocampal neurons, fetal hippocampal cell grafts, neuronal precursor cells from medial ganglionic eminence, and other neural stem cells (NSCs) have been tested as efficacious transplantable cells in epilepsy models [14–18]. For replacement of GABAergic neurons, embryonic stem cell-derived GABAergic neurons also show solid efficacy data in epileptic animals [19,20]. Very recently, GABAergic inhibitory interneurons transplanted into the amygdala rescued the hyperactivity deficit of epilepsy model of mice, but not seizure activity or other abnormal behaviors, although transplantation into the hippocampus improved hyperactivity as well as seizure activity and other abnormal behaviors, which indicates the important roles of interneurons for epilepsy therapy [21]. Among the many mechanisms of action suggested to mediate the therapeutic benefits of stem cell therapy in epilepsy involve the ability of the transplanted cells to promote neuroprotective/neurorestorative effects against the damaged hippocampal tissue through direct and/or indirect effects on neurons, such as promoting neurogenesis, angiogensis, synaptogenesis, and anti-inflammatory effects [22,23].

In our laboratory, adult-derived NSCs were implanted into the hippocampus of rats receiving intraventricular KA administration [22]. At 2 weeks after KA administration, rats receiving NSC transplantation demonstrated decreased abnormal spikes as evidenced by electrophysiological recordings. At 5 weeks after transplantation, surviving grafts were detected around the CA3 and migrated into the subgranular zone. Some transplanted cells expressed NeuN, which is phenotypic marker for mature neurons, although most transplanted cells expressed the astrocytic marker, glial fibrillary acidic protein. Immunohistochemically, abnormal mossy fiber sprouting was suppressed and GABAergic inhibitory neurons were preserved in the transplanted rats. In addition, transplanted NSCs were shown to secrete trophic factors, including stem cell factor [24]. A recent study demonstrated that stem/progenitor cells positive for c-kit, a ligand of stem cell factor, increased the expression level of glutamate transporter GLT 1 in the surrounding astrocytes; subsequent lowering of extracellular glutamate level was shown to reduce cell damage to surrounding tissue [25]. Other than that, NSCs might enhance endogenous neurogenesis to repair damaged tissue. Thus, stem cell therapy for epilepsy may supplement the diseased brain with pro-survival growth factors and subsequently improve the host microenvironmental niche for the spared host intrinsic neurons that altogether may bring about suppressed hyperexcitability of the abnormal neuronal circuits, as well as replacement of damaged neurons.

Along this vein of supplying neurotrophic factors to the epileptic brain, we explored the utility of encapsulated cell transplantation [26]. The advantages of this method are as follows: (1) Various neurotransmitters or neurotrophic factors can be produced continuously from encapsulated cells with tailored properties. Cells inside the capsule survive with sufficient nutrient and oxygen through the semipermeable membrane and capable of secreting factors outwards; (2) Minimal immune reactions and even lack of immunological rejection arise because cells are inside and protected by capsule. The molecular sieve prevents immunocompetent cells from invading into the capsule and attacking the donor cells; (3) Tumorigenesis does not occur because donor cells stay inside the capsule; (4) The capsule can be removed from the transplanted brain in case adverse effects arise after transplantation; (5) Various cells including immortalized cell lines and xenografts can be transplanted safely as donor cells. Compared to the direct neurotrophic factor infusion using a minipump system, the delivery of freshly secreted factors with no degradation and the host-graft interaction that may allow a dynamic regulation of growth factor secretion are the advantages of encapsulated cell transplantation. As cell sources for neurotrophic factors, mesenchymal stem cells (MSCs) and umbilical cord blood cells have been demonstrated to possess robust secretory functions [27,28].

In parallel to encapsulated cell transplantation for epilepsy, non-encapsulated transplant studies have also been tested in epilepsy models. Genetically-engineered MSCs were promoted to differentiate into GABAergic neurons and transplanted into epileptic rats with consequent functional amelioration [29]. Another recent study using MSC transplantation for epileptic animals shows attractive results [23]. MSCs were transplanted at 3 weeks and 10 months after pilocarpine injection. In both experiments, MSCs exerted neuroprotective/neurorestorative effects with anti-inflammatory effects. Interestingly, doublecortin positive neuronal precursor cells decreased in rats receiving MSCs at 3 weeks after insult, but the number of doublecortin positive cells increased in rats receiving MSCs at 10 months after insult, compared to control rats receiving saline injection. Because abnormal neurogenesis accompanies epileptogenesis in the acute phase after insult, the reduced neurogenesis (albeit epilepsy-induced abnormal cell proliferation) would be considered beneficial. In the chronic phase, however, neurorestorative neurogenesis is a key to rescuing against neurodegeneration inherent in epilepsy, and this can be achieved by MSC transplantation. The need for this dynamic suppression and enhancement of neurogenesis in epilepsy highlights the requirement for a cross-talk, either at the cellular or the molecular level, between the transplant and the host brain. This interesting topic on epileptogenesis and neurogenesis was reviewed previously [30], although controversy remains on the pathological and therapeutic consequences of this phenomenon. Notwithstanding that the interaction between epileptogenesis and neurogenesis may be dictated by the surrounding environment, the timing, and its corresponding degenerative and restorative processes after the insult.

Recent development in pluripotent stem cells for diseases in the central nervous system was fast-evolving [31]. A recent study demonstrated a highly efficient method to induce human pluripotent stem cells into primitive neural stem cells in 7 days. Subsequently, they achieved the differentiation of neural stem cells into region-specific neuronal subtypes, such as GABAergic, dopaminergic, and motor neurons [32]. Thus, the time- and labor- saving methods of neural differentiation might contribute to epilepsy therapy. For a breakthrough in epilepsy therapy, patient-derived iPS cells might play a critical role. GABAergic neurons were isolated from patients of Dravet syndrome, which is a devastating infantile-onset epilepsy syndrome with cognitive deficits and autistic traits caused by genetic alterations in sodium channels. The dysfunction of inhibitory GABAergic neurons was confirmed electrophysiologically, which seems to be a main driver in epileptogenesis [33]. Another group showed hyperexcitability of iPS cells-derived GABAergic neurons and alleviation of hyperexcitability of it by administration of phenytoin, a conventional anti-epileptic drug [34]. These methods might be potentially used for screening appropriate drugs for personalized therapies and for revealing the underlying mechanisms of epileptogenesis.

#### 2.2.2. Neuroprotective Agents

Many neurotrophic factors have been explored as therapeutic modalities against TLE. Intrahippocampal overexpression of fibroblast growth factor 2 and brain-derived neurotrophic factor (BDNF) resulted in decreased neuronal cell death against pilocarpine-induced status epilepticus through enhanced neurogenesis and anti-inflammatory effects [35]. Similarly, administration of insulin-like growth factor 1 exerts neuroprotective and anti-inflammatory effects on KA-induced animal models of TLE which coincided with improved cognitive function [36]. Additional growth factor-based therapies include altering the mammalian target of rapamycin (mTOR) signaling pathway, which led to a novel pharmacological modulation of epileptogenesis [37]. Disagreement exists on whether BDNF accelerates epileptogenesis with consequent seizure attacks or exerts anti-epileptic effects. BDNF binds to the receptor tyrosine kinase, TrkB. Increased BDNF-TrkB signaling might facilitate epileptogenesis [38]. A Review of BDNF-TrkB signaling reveals that seizures induce remarkable increase of BDNF expression with enhanced activation of TrkB in the mossy fiber pathway of hippocampus [39]. Moreover, intraventricular infusion of BDNF (1 or 3 μg/h for 1 week) led to spontaneous epilepsy [40]. Similarly, overexpression of BDNF increased seizure severity [41]. In parallel, matrix metalloproteinase-9 was shown to aid in epileptogenesis by converting proBDNF to mature BDNF in the hippocampus, which also supports that BDNF accelerates epileptogenesis. On the other hand, several studies revealed anti-epileptic effects of BDNF [35]. Based on our studies, we confirmed that continuous administration of BDNF at low dose exerted anti-epileptic effects using encapsulated BDNF-producing cell transplantation as described in the prior section [42]. In the study, the capsule produced about 200–300 pg of BDNF per hour. Results revealed behavioral and electrophysiological ameliorations in the BDNF-treated group. Immunohistochemically, the number of doublecortin positive neuronal precursor cells increased in the dentate gyrus with more preserved NeuN positive neurons in the CA1 and CA3, compared to that of control rats. Continuous intrahippocampal administration of BDNF likely attenuated the development of epilepsy mainly through increased expression of neuropeptide Y (NPY) [43]. These studies caution that the dose and timing of BDNF administration are critical for epilepsy especially in view of BDNF being upregulated in the hippocampus of epileptic patients suggesting that large amount of exogenous BDNF may worsen the epilepsy.

Recent studies have also evaluated the therapeutic efficacy of erythropoietin (EPO). EPO is a well-known hematopoietic hormone with demonstrated neuroprotective effects for diseases in the central nervous system, including cerebral ischemia and Parkinson’s disease [44,45]. In febrile seizure models, EPO exerted anti-epileptic effects through anti-inflammatory effects and molecular alterations after seizures [46]. In our laboratory, intraventricular EPO infusion significantly ameliorated behavioral scores and surviving rates of rats receiving intraperitoneal injection of KA. NeuN positive neurons in the CA1 were significantly preserved through anti-apoptotic effects. Furthermore, abnormal neurogenesis was suppressed. Those anti-epileptic effects were cancelled by administration of NPY Y2 receptor antagonist, which indicates that therapeutic effects of EPO against epilepsy were mediated at least partially by NPY [47]. A recent study demonstrated that intraventricular NPY infusion exerted neuroprotective and neurogeneic effects against experimental epilepsy [48]. In another study of ours using the combined therapy of NSC transplantation and EPO infusion, EPO strongly enhanced the surviving rate of transplanted NSCs [10]. Surprisingly, the combined therapy significantly reduced mossy fiber sprouting, compared to other groups [10]. Furthermore, our recent data suggest robust neuroprotective effects, but without hematopoietic consequences, by carbamylated EPO Fc fusion protein in a Parkinson’s disease model [49]. We envision that this protein may be a new therapeutic agent for epilepsy.

#### 2.2.3. Electrical Stimulation

Compared to the two therapeutic modalities described above, electrical stimulation seems more realistic largely based on its long history of medical use. Vagal nerve stimulation has been routinely used for refractory epilepsy [50,51]. Electrical stimulation is relatively safe and offers reversibility, but the therapeutic outcomes are limited and the financial cost to the patient is quite expensive. Mechanistic-wise, the direct effects of vagal nerve electrical stimulation may be exerted in several regions of brain via neuronal structures surrounding the stimulated tissue including raphe nuclei and locus ceruleus [52,53]. Vagal nerve stimulation induced slow hyperpolarization in neurons of cerebral cortex [54] and elevates seizure threshold in the kindling model [55]. Electrical stimulation for two days caused about 50% increase of BrdU positive cells in the hippocampus [56]. Furthermore, vagal nerve stimulation for one month enhanced expression of BDNF in the CA3 with morphological changes of newly developed neurons [57]. These studies indicate that vagal nerve stimulation may directly alter the epileptogenesis via phenotypic cellular reorganization within the core brain tissue source of the seizure.

Other than vagal nerve stimulation, deep brain stimulation, epidural stimulation, and transcranial magnetic stimulation have been assessed in epilepsy, but with controversies. In our previous study, anterior thalamic nucleus was chemically suppressed in amygdala-kindled rats [58]. Subsequently the treated rats showed significant behavioral ameliorations with reduced abnormal neurogenesis in the hippocampus. Anterior thalamus may be a good target of electrical stimulation for epilepsy, in that the suppression of anterior thalamic activity seems to limit the propagation pathways for generalized seizures [59]. Alternatively, the substantia nigra pars reticulata, subthalamic nucleus, cerebellum and cerebral cortex may be other targets for electrical stimulation when contemplating with arresting seizure propagation against epilepsy [60–62].

Recently, we generated new data on deep brain stimulation and epidural electrical stimulation for stroke animals and provided evidence of neuroprotective/neurorestorative effects [63,64]. The mechanisms underlying the observed therapeutic benefits involve neurogenesis, angiogenesis, increased secretion of neurotrophic factor/growth factors, anti-inflammatory effects, and anti-apoptotic effects through phosphoinositide 3-kinase pathways. Of note, we showed that electrical stimulation at low frequency exerted therapeutic effects. Similarly, spinal cord electrical stimulation has been shown as an alternative therapeutic option for Parkinson’s disease and that stimulation at low frequency enhanced neuronal plasticity [65]. In consideration of these several line of investigations, further exploration of electrical stimulation will likely open new avenues for development of treatments for epilepsy. In tandem, our better understanding of the disease pathology, especially cell death and survival mechanisms, will equally aid in designing novel therapeutics for epilepsy and related neurological disorders.

## 3. Conclusions

Epilepsy treatment has traditionally involved antiepileptic drugs and subsequently has been explored using surgical approaches including electrical stimulation. The progress in this field has been facilitated by the standardization of epilepsy animal models. The role of basic research for epilepsy is essential. Translational research has reached the stage for efficacy and safety readouts to facilitate the entry of novel therapies to the clinic. Regenerative medicine for epilepsy is an exciting and challenging new area which may reap cutting edge discoveries in our knowledge about disease pathology, its mechanisms and treatments. We envision that cell therapy, novel neuroprotective agents, and electrical stimulation epitomize the new frontiers of regenerative medicine for epilepsy, and basic and translational investigations along these lines should be considered to facilitate their clinical applications in epileptic patients, although these methods require neurosurgical procedures.
